# Application of a Decision Tree Model to Predict the Outcome of Non-Intensive Inpatients Hospitalized for COVID-19

**DOI:** 10.3390/ijerph192013016

**Published:** 2022-10-11

**Authors:** Massimo Giotta, Paolo Trerotoli, Vincenzo Ostilio Palmieri, Francesca Passerini, Piero Portincasa, Ilaria Dargenio, Jihad Mokhtari, Maria Teresa Montagna, Danila De Vito

**Affiliations:** 1 School of Specialization in Medical Statistics and Biometry, School of Medicine, University of Bari Aldo Moro, 70121 Bari, Italy; 2 Department of Interdisciplinary Medicine, University of Bari Aldo Moro, 70121 Bari, Italy; 3 Department of Biomedical Science and Human Oncology, University of Bari Aldo Moro, 70121 Bari, Italy; 4 Department of Basic Medical Sciences, Neurosciences, and Sense Organs, Medical School, University of Bari Aldo Moro, 70121 Bari, Italy

**Keywords:** COVID-19, machine learning, clinical aspect, prognostic markers, haematochemical parameters, prediction

## Abstract

Many studies have identified predictors of outcomes for inpatients with coronavirus disease 2019 (COVID-19), especially in intensive care units. However, most retrospective studies applied regression methods to evaluate the risk of death or worsening health. Recently, new studies have based their conclusions on retrospective studies by applying machine learning methods. This study applied a machine learning method based on decision tree methods to define predictors of outcomes in an internal medicine unit with a prospective study design. The main result was that the first variable to evaluate prediction was the international normalized ratio, a measure related to prothrombin time, followed by immunoglobulin M response. The model allowed the threshold determination for each continuous blood or haematological parameter and drew a path toward the outcome. The model’s performance (accuracy, 75.93%; sensitivity, 99.61%; and specificity, 23.43%) was validated with a k-fold repeated cross-validation. The results suggest that a machine learning approach could help clinicians to obtain information that could be useful as an alert for disease progression in patients with COVID-19. Further research should explore the acceptability of these results to physicians in current practice and analyze the impact of machine learning-guided decisions on patient outcomes.

## 1. Introduction

A new form of pneumonia spread in Wuhan, Hubei Province of the People’s Republic of China, beginning in December 2019, from an unidentified microbiological agent [1] later identified as a novel coronavirus called severe acute respiratory syndrome coronavirus 2 (SARS-CoV-2) [2], and the resulting disease was named coronavirus disease 2019 (COVID-19). Since the end of 2019, the outbreak of COVID-19 has spread worldwide. As of 1 January 2021, there had been 2,123,776 confirmed cases and 74,261 deaths.

The initial symptoms of SARS-CoV-2 infection are similar to those of influenza but vary from person to person; they can be asymptomatic, paucisymptomatic, or symptomatic. The symptoms are fever, tiredness, anorexia, headache, diarrhea, sore throat, mild dyspnea, malaise, blocked nose, nausea, and vomiting [3]. People with pre-existing comorbidities (chronic obstructive pulmonary disease (COPD), hypertension, obesity, diabetes, heart disease, liver disease, acquired immunodeficiency syndrome (AIDS), renal disease, and cancer) have an increased risk of death or more critical COVID-19 [4,5]. An increase in the incidence and prevalence of COVID-19 has led to an increase in hospitalizations worldwide, and the most severe cases are characterized by acute respiratory syndrome, requiring hospitalization in intensive care units (ICU) [6,7].

ICU resources in hospitals have been stressed, especially if they lack adequate facilities, staff [8], and global health services for related and unrelated COVID-19 diseases [9]. The mortality of patients with severe SARS-CoV-2 pneumonia has been and is still considerable, especially in patients older than 65 years and with relevant comorbidities [6,8].

The need to create a model that would allow the detection of the main characteristics of severe disease and identify features that could predict the outcome of COVID-19 to better manage patients’ health as well as economic resources was soon felt [10].

Therefore, many studies have been conducted worldwide on patients with SARS-CoV-2 that consider patients’ demographics, clinical symptoms and signs, and blood chemistry data to predict the outcomes of death or ICU admission [10,11]. Different study designs were used to evaluate predictors, as retrospective studies mainly focused on immunological features [12] or clinical and laboratory values. Descriptive studies were also proposed in the initial phase of the pandemic to define signs and symptoms in patients [13].

A meta-analysis published in 2020 summarized the main biomarkers to monitor patients with COVID-19 [14]. Hematological biomarkers included white blood cells, neutrophils count, lymphocytes count, monocytes count, eosinophils count, platelet count, cluster of differentiation (CD)4, CD8 percentages, and hemoglobin. Biochemical markers were albumin, alanine aminotransferase, aspartate aminotransferase, total bilirubin, creatinine, creatinine kinase, lactate dehydrogenase (LDH), cardiac troponin I, myoglobin, and creatine kinase-MB. The coagulation markers were prothrombin time, activated partial thromboplastin time (APTT), and D-dimer. The inflammatory biomarkers were C-reactive protein (CRP), serum ferritin, procalcitonin (PCT), erythrocyte sedimentation rate, and interleukin and tumor necrosis factor-alpha (TNFα) levels. 

Another retrospective study proposed a clinical risk score for critically ill patients with COVID-19, which was validated by including clinical symptoms, signs, and laboratory tests [15]. Furthermore, the results of the studies have been valuable for blood chemistry tests; white blood cells, neutrophils, lymphocytes, monocytes, eosinophils, platelets, CRP, D-dimer, and PCT were analyzed [16,17].

The main objective of this study was to evaluate, in a prospective cohort of COVID-19 patients hospitalized in a non-ICU medicine ward, the application of a decision tree to predict bad outcomes (death or transfer to an intensive care unit) and the relationships between main demographic, clinical, blood chemistry, and immunological features. The method chosen to determine valuable predictors is a machine learning approach based on a decisional tree. This assesses an algorithm and finds a useful threshold, when appropriate for continuous variables, that could guide clinicians in weighting the value of each feature in the evaluation of the clinical course.

## 2. Materials and Methods

### 2.1. Participants and Procedures

A prospective observational study was performed during the pandemic period from 2 January to 30 April 2021, including 146 consecutive not-vaccinated patients admitted to a dedicated internal medicine COVID unit (COVID-MI) in the large regional hospital Policlinico of Bari, Apulia. One patient was removed from the analysis because of missing data both on outcome and predictor variables; therefore, a total of 145 patients were included in the final analysis. Patients arrived from the emergency room after a confirmed diagnosis of infection with SARS-CoV2 by a positive rinopharyngeal swab. Data were collected upon admission and after 10 days. The outcome was registered from clinical documentation and was defined as the combined endpoint of death or transfer to ICU, whichever was first.

The main clinical symptoms, anamnestic conditions, blood exams, and immunological panels were taken: blood pressure, respiratory frequency, cardiac frequency, temperature, O_2_ saturation, red blood cell count, hemoglobin, neutrophil count, lymphocyte count, platelet count, serum C-reactive protein, procalcitonin, lactic dehydrogenase (LDH), albumin, aspartate aminotransferase (AST), alanine aminotransferase (ALT), bilirubin, alkaline phosphatase (ALP), creatine kinase (CPK), serum sodium (Na), serum potassium (K), serum chlorine (Cl), D-dimers, international normalized ratio (INR), interleukin-6 (IL-6), immunoglobulin M, and G (IgM and IgG) against SARS-CoV-2. 

Temperature was detected by a clinical ecologic axillary thermometer. Saturation and other clinical parameters were detected by a modular multiparameter monitor (Life Scope VS, Nihon Kohden, Japan). This study was approved by the Interregional Ethical Committee of Azienda Ospedaliero Universitaria Consorziale Policlinico.

### 2.2. Statistical Analysis

Quantitative data are presented as median and interquartile range (IQR) because after the evaluation of normal distribution by the Shapiro–Wilk test, data were found to be not normally distributed. Comparison between independent groups was performed using the Mann–Whitney test.

Categorical variables are summarized as counts and percentages, and comparisons between independent groups were performed by the chi-square test or Fisher exact test as appropriate.

Statistical significance was set at *p*-value < 0.05.

To identify variables that could be valuable predictors of the outcome, a machine learning (ML) method based on the Quinlan boosting C5.0 algorithm that uses a confidence factor (CF) set at 0.25 to assign an observation to a class was applied. 

Other hyperparameters (winnowing, boosting, iteration) were chosen by the application of the “one standard error rule” that consists of selecting variables that define a model within one standard error of the minimum cross-validation error. 

The variables used in the model for the training set were as follows: sex (male or female); presence of symptoms of infection (nasal congestion, headache, tussis, pharyngodynia, dyspnea, fever, and myalgia); presence of chronic pulmonary disease, mainly COPD; diabetes; hypertension; cardiovascular diseases; cerebrovascular diseases; hepatitis B and C; tumors; chronic kidney diseases; and immunopathological diseases. Continuous variables included age, cardiac frequency, oxygen saturation, blood pressure, fraction of inspired oxygen (FiO_2_), temperature, neutrophils, lymphocytes, platelet, hemoglobin, C-reactive protein (CRP), LDH, albumin, AST, ALT, ALP, bilirubin, creatinine, CPK, Na, K, Cl, D-dimers, INR, and IgM and IgG antibodies against SARS-CoV-2. Data from the second blood collection were analyzed separately using the same method.

To evaluate the accuracy, a k-fold cross validation method with repetition was applied. The set was split in subsets as defined by the parameter “k”, and the procedure was then repeated. We chose the default values k = 10 and repetition = 10. 

The measures of accuracy were based on the area under the ROC curve (AUC) and on the indicators suggested by Iwendi [18] and Bottino [19], determining sensitivity (true positive divided all events), specificity (true negative divided all non-events), positive predictive values (true positive divided all predicted as events), negative predictive value (true negative divided all predicted as non-events), global accuracy (the sum of true positive and true negative divided the whole sample), F1-score (the ratio of twice the product between precision and recall divided the sum of precision and recall), Matthew correlation coefficient (MCC), and balanced accuracy (the mean of sensitivity and specificity). Collinearity was evaluated on variables entered in the model determining the VIF (variance inflation factor) and tolerance, using as a threshold VIF > 5 and tolerance < 0.25.

### 2.3. Software

The model was run in R-Studio version 1.4.1106 [20] using, for data arrangement and description of the output used, the following packages: C5.0 [21], caret [22] and pROC [23]. Data management and descriptive statistics were performed using SAS/STAT version 9.4 for PC (SAS Institute, Cary, NC, USA).

## 3. Results

**Figure 1:**
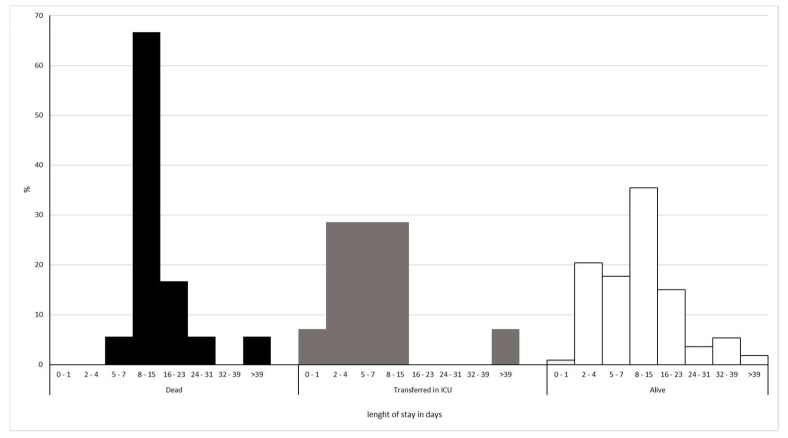
Percentage distribution of patients by length of stay in days and outcome.

The median age of the patients was 71 years (IQR 58–82), and 54.8% were male. The main characteristics of the patients are summarized in Table 1 and Table 2. The outcome, as a combined endpoint of death and transfer to the ICU, was observed in 22.1% (32/145) of patients, and death occurred in 56.3% (18/32) with a median LOS (length of stay) of 13.5 days (IQR 9-18). Transfer to the ICU occurred in 43.7% (14/32), with a median LOS of 5.5 (IQR 3.25-12). There were 113 patients discharged alive with a median LOS of 9 days (IQR 5-16) (Figure 1). There were statistically significant differences in the comparison between dead vs. ICU transferred patients (*p* = 0.006).

The comparison of blood analysis and other main clinical characteristics measured after the admission in the COVID-MI ward between patients discharged alive and those with negative outcomes (deaths or transferred in ICU) are shown in Table 2. 

**Figure 2:**
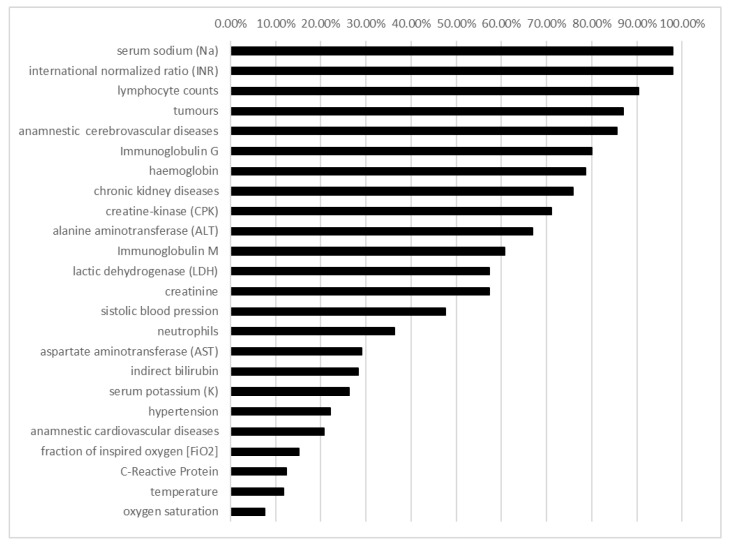
Variables’ importance (attribute usage) for the training decision tree model C5.0.

The variables used in the model are shown in Figure 2. The most important predictors in the model’s training were the IgG and IgM values that reached 100% attribute usage. Age was a predictor of 100% attribute usage. Sex, CPK, CRP, platelet count, LDH, K, NA, INR, D-dimers, ALT, AST, creatinine, hemoglobin, and neutrophil and lymphocyte counts had an attribute usage of 90–99%. 

**Figure 3:**
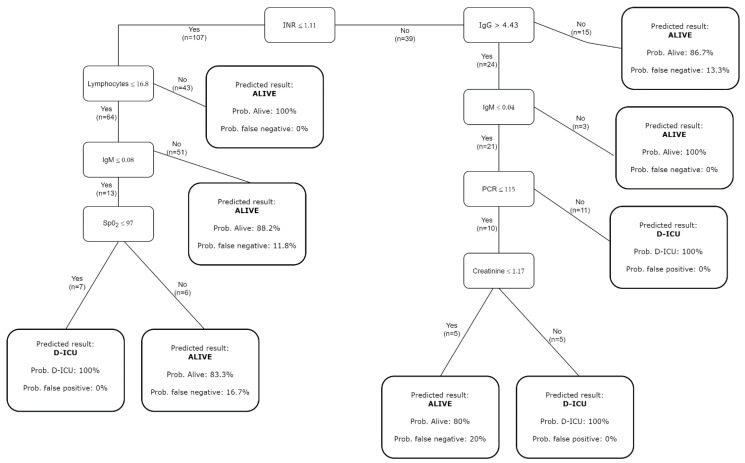
Graphical representation of the decision tree model built on the training dataset with the predicted outcome and the fitting goodness (probability of correct outcome and its complementary value).

To predict the outcome (Figure 3), the decision tree started with the value of INR, and a cut-off was defined as equal to or lower than 1.11 and greater than 1.11. Thus, we had two branches to classify patients: the first uses lymphocytes, IgM, and oxygen saturation; the second uses IgG, IgM, CRP, and creatinine.

The outcome D-ICU for patients with INR ≤ 1.11 was predicted after three consecutive steps: lymphocyte ≤ 16.8 × 10^3^, IgM ≤ 0.08 AU/mL, and oxygen saturation ≤ 97%. The prediction for patients with INR > 1.11 was achieved after four steps with IgG ≤ 4.43 AU/mL, IgM ≤ 0.04 AU/mL, CRP ≤ 115 mg%mL and creatinine ≤ 1.17 mg%mL. The error on the training set for all previous classifications was 0%, indicating that the decision tree correctly classified all patients who died of ICU transfer.

The variables entered in the model should not be affected by collinearity; they have all shown a VIF < 5 (the higher was 1.28 for IgG) and tolerance >0.25 (the lower was 0.78 for IgG).

**Figure 4:**
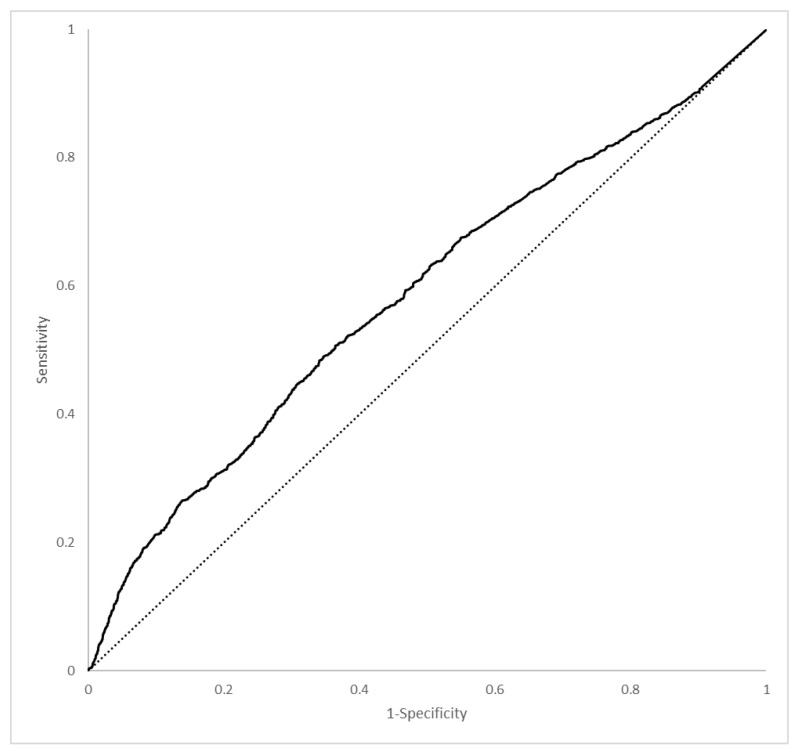
Receiver operating characteristic curve to evaluate the fitting of the model.

The fitting of the model was evaluated on the validation set which showed a total accuracy of 75.93%, balanced accuracy of 61.52%, and sensitivity and specificity of 99.61% and 23.43%, respectively (Table 3). The value of the F1-score to evaluate the fitting of the model was 89.17, and the MCC was 17.94%; both were low as the area under the ROC curve (Figure 4) was 0.61.

The follow-up values of biochemical and hematological variables were available for only 28 patients, but neither the difference between the first and second data collection nor the value of the second data collection allowed a model that could predict the outcome.

## 4. Discussion

This prospective study, conducted during the pandemic wave in a COVID-19 medicine ward, has allowed us to identify predictors of outcome using a machine learning algorithm. The decision tree model showed the sequence and threshold for each variable to predict unfavorable outcomes. 

Various artificial intelligence algorithms have been used to predict death or hospitalization in the ICU following COVID-19 [24,25]. Among the various machine learning models, the most widely used for classification are XGBoost, linear regression, support vector machine, decision tree, random forest, and neural network convolution. For an easier understanding and representation of our study, we used a C5.0 decision tree model as an algorithm. This algorithm is one of the most commonly used approaches for representing recursive classifiers [26]. The choice of method has a consequence on the accuracy. The study by De Souza [25] compared the accuracy among models, and the decision tree had the same results with respect to XGBoost, naive Bayes, and support vector machine. Interestingly, machine learning based on logistic regression is more accurate. The decision tree algorithm split sets of data recursively, considering the symptoms; however, many other parameters predict SARS-CoV-2 infection until the procedure reaches its maximum depth [25,27].

The values of the indicators to evaluate the validity of the model have appeared as promising, given the 99.61% sensitivity and the 75.93% global accuracy. The AUC from the ROC analysis resulted in a small value of 0.61. This suggests that our variable could have a low prediction accuracy, even if we detected all clinical parameters at the entrance of the patients into the medicine unit. The clinical condition at the beginning of hospitalization could not be enough to predict the outcome. The search for a model considering clinical characteristics at the entrance to the non-intensive medical unit is an important issue. Our data show that changes in patients happen quickly given that the median of transfer to ICU was 5 days and the median of days to death was 13 days. Therefore, the search for conditions that could help to understand the course of the disease is a critical issue.

Comparing our model with other decision tree models, it can be observed that our model has an accuracy similar to that of Migriño (81.5%) [28], but lower than that of Altini et al. [29] and Naseem et al. [30], which were both higher than 85%. It is noteworthy that our model specificity was low when compared to other research [29,30]. On the other hand, the model has a 89,16% F-score, which is higher than another machine learning model that predicted outcomes in patients with COVID-19 [28]. Souza [25] has shown that this score is the lowest among the algorithms, and it was 52% in the training and 38% in the test set. 

According to our model, the INR value is the first criterion used to classify patients. The INR is a parameter that more accurately evaluates the prothrombin time (PT) to eliminate the inherent variability in the calculation between various laboratories. It is calculated by dividing the PT of each patient by a standard laboratory parameter [31]. The INR value was lower among participants who survived during hospitalization than those who died or were transferred to the ICU. The difference was statistically significant (*p* < 0.001). Other studies have shown an increase in the INR value in participants with poor outcomes during hospitalization compared to those who survive [30,32,33]. INR assumes an important prognostic value as it is representative of the activation of coagulation, and it is now well known that COVID-19 is associated with coagulopathy [34]. Infection with SARS-CoV-2 can damage the vascular endothelium (typically at the pulmonary level and then in the subsequent phases at the systemic level) and initiate an inflammatory process that can alter the normal homeostatic procoagulant pathways and anticoagulants [35]. Our cohort had a high percentage of patients older than 70 years with cardiac or cerebrovascular disease who could be treated with anticoagulation therapy. In a practical application of the results of this model, the information coming from INR should be evaluated in light of previous therapies, even though the model did not consider age and comorbidities as valuable help.

A significant difference in lymphocyte and neutrophil counts was not found in our study between patients who survived and those who died or were transferred to the ICU; however, it resulted in an important variable in the decision tree model. Peripheral lymphocyte count is an early indicator of severe or critical COVID-19 patients [36], and in a previous study conducted by Naseem et al. [30] using a Cox regression model, an increased risk of death was associated with lymphocyte count reduction.

Serum creatinine was found as a significant variable to predict a bad outcome, and that parameter was found to be a significant variable to predict mortality in a meta-analysis of 19 studies on COVID-19 patients [37]. This sign of kidney damage should be given more attention because it is associated with worse outcomes in older and younger patients [38].

The comparison of IgM values, IgG levels and age between the patients who died or were transferred to the ICU and the surviving patients showed significantly different median values (*p* = 0.032 for IgM; *p* = 0.023 for IgG). Moreover, they are the parameters most used by the machine learning C5.0 algorithm for the construction of the decision tree. To the best of our knowledge, only a few other authors have used blood immunoglobulin levels to predict patient outcomes. In a retrospective study, Suryawanshi et al. [39] found that IgG concentration was different in three groups of patients classified according to severity. No difference was found in IgM concentration. In parallel, Yuan et al. [40] found a difference in IgG-only concentrations between non-serious patients and those with severe or critical conditions.

In a recent paper, some of us showed a reduced mortality in patients affected by COVID-19 infection linked with early antibodies against SARS-CoV-2, irrespective of age [41]. The authors actually demonstrate that an efficient immunological response during the early phase of COVID-19 protects from mortality, irrespective of age, even if advanced age is a critical risk factor for a poor outcome in infected subjects. These results are consistent with the data of the present work, since the levels of both IgM and IgG anti-SARS-CoV-2 in our model have a critical role in the prediction of the evolution of the disease. Possible therapeutic interventions able to enhance humoral immunity in elderly patients with weak antibody response during the early stage of COVID-19 infection are warranted.

## 5. Study Limitation

A limitation of this study is the intrinsic fault of the decision tree model related to overfitting, which often occurs in complex models for relatively simple data [42]. This could affect our results in which we have found low values of false negatives. 

The decision tree model does not generate explicit coefficients, such as those generated using a logistic regression model. Therefore, it is difficult to estimate the impact of each variable on the outcome in terms of risk, but it is possible to have information on useful variables and their decisional values in a way that could be familiar to a clinical audience.

Finally, our model was trained on the data of a single cohort; therefore, if it is to be used outside of this, it is advisable to carry out independent validation and possible requalification of the model.

The unknown effect of sample size on the effectiveness of results: the study was planned for a larger sample size, but difficulties related to hospitalization overload determined the uneasy dialogue with clinicians to collect data. A simulation to determine sample size and power for a logistic regression to evaluate the effect of a single variable with an odds ratio of 2, and the probability of the event given the presence of the risk factor, requires a sample size of 178 subjects. However, there are still debatably reliable methods to determine sample size for machine learning methods that work with reliable results on so called “big data”, and sample size determination for decision trees or other algorithms require proper methods.

## 6. Conclusions

A machine learning approach, the decision tree model, was used to analyze the clinical data of hospitalized COVID-19 patients to establish an efficient prognosis [29]. In this study, we used the clinical, demographic, and blood chemistry parameters of the patients in order to predict two possible outcomes: discharged alive, or transferred to ICU or death, whichever was first.

The results suggest that a machine learning approach could help clinicians evaluate disease conditions in patients with COVID-19, and it could be useful in guiding decisions for therapies or diagnostic procedures. Further research should explore the acceptability of these results to physicians in current practice and analyze the impact of machine learning-guided decisions on patient outcomes, such as the feasibility of using the selected information and cut-offs.

We say that the study is prospective, but the machine learning model is a post hoc theoretical application. Therefore, we believe that in future studies, it could be introduced in the management of patients affected by COVID-19 to see how it affects the decision process of clinics in real practice. 

We also believe it is necessary that further studies be conducted to evaluate machine learning models to better understand the identified predictors, especially those of an immunological nature, which are still poorly analyzed. Furthermore, more complex models, such as artificial neural networks and deep learning models, should be implemented together with an easier system to interpret whether the result is useful for clinical decisions [43].

## Figures and Tables

**Table 1:** Main characteristics of the patients included in the study at baseline and results of comparison of percentage between outcome using chi-square or Fisher exact test.

	Death or Transferred to Intensive Care Unit (*n* = 32)		Discharged Alive (*n* = 113)		
	N	%	N	%	*p*-Value
**Sex**					
Male	18	56.25%	61	53.98%	1.00
Female	14	43.75%	52	46.02%	
**Symptoms**					
Dyspnea	12	37.50%	52	46.02%	0.999
Cough	5	15.63%	35	30.97%	1.00
Fatigue	7	21.88%	30	26.55%	1.00
Headache	2	6.25%	12	10.62%	1.00
Confusion	1	3.13%	9	7.96%	1.00
Nausea	1	3.13%	8	7.08%	1.00
Sick	1	3.13%	6	5.31%	1.00
Pharyngitis	1	3.13%	6	5.31%	1.00
Nasal congestion	1	3.13%	3	2.65%	0.999
Arthralgia	0	0.00%	3	2.65%	1.00
Myalgia	1	3.13%	2	1.77%	0.997
Arrhythmia	3	9.38%	12	10.62%	1.00
**Comorbidity**					
Hypertension	12	37.50%	71	62.83%	0.356
Cardiovascular disease	12	37.50%	43	38.05%	1.00
Diabetes	11	34.38%	35	30.97%	1.00
Cerebrovascular disease	9	28.13%	19	16.81%	0.896
Chronic kidney disease	8	25.00%	14	12.39%	0.585
COPD	5	15.63%	14	12.39%	0.999
Tumors	5	15.63%	11	9.73%	0.986
Hepatitis B	0	0.00%	6	5.31%	0.974
Immunopathological disease	1	3.13%	5	4.42%	1.00

**Table 2:** Comparison between patients discharged alive and those who died or were transferred to an intensive care unit by demographical, and baseline clinical, hematological, and biochemistry value. The *p*-value refers to result of Wilcoxon test for independent groups.

	Patients Deaths or Transferred in ICU (*n* =32)			Patients Alive (*n* = 113)			*p*-Value
	Median	Q1	Q3	Median	Q1	Q3	
Age (years)	78.0	67.0	85.75	70.0	57.0	82.0	0.011
Temperature (°C)	36.5	36.0	36.6	36.4	36.2	36.67	0.715
Respiratory rate (rpm)	20.0	18.0	20.0	18.0	15.0	20.0	0.110
Cardiac frequency (bpm)	79.0	70.0	99.0	82.5	75.0	92.5	0.515
Systolic blood pressure (mmHg)	137.5	116.0	150.0	130.0	122.5	145.0	0.947
Diastolic blood pressure (mmHg)	77.of 5	65.0	82.5	77.0	70.0	85.75	0.643
Temperature at admission (°C)	36.5	36.0	36.6	36.4	36.2	36.67	0.715
Percentage of O_2_ saturation	97.0	94.75	97.25	97.0	96.0	99.0	0.017
FiO_2_ (%)	50.0	28.0	80.0	37.5	21.0	60.0	0.227
Neutrophil count (×10^3^%µL)	79.8	74.525	85.32	77.4	69.25	84.4	0.124
Lymphocyte count (×10^3^%µL)	13.4	8.9	16.35	14.95	9.4	21.3	0.115
Platelet count (×10^3^%µL)	202,000	147,250	272,250	222,500	168,500	292,000	0.212
Hemoglobin level (g%dL)	127.0	116.75	143.0	123.0	108.0	137.0	0.162
Procalcitonin levels (ng%mL)	0.11	0.09	0.27	0.12	0.06	0.23	0.712
CRP (mg%mL)	79.7	30.6	105.5	37.6	14.0	97.6	0.066
LDH (mg%mL)	307.0	258.75	368.0	256.0	207.0	309.0	0.007
Albumin (mg%mL)	27.0	24.0	30.5	28.0	25.0	31.0	0.098
ALT (mg%mL)	23.0	14.75	52.0	27.0	20.0	47.0	0.413
AST (mg%mL)	30.0	22.0	48.0	28.5	21.0	38.5	0.371
ALP (mg%mL)	72.0	58.5	87.0	64.0	53.0	83.0	0.441
Direct bilirubin (mg%mL)	0.01	0.009	0.0168	0.01	0.007	0.0139	0.041
Indirect bilirubin (mg%mL)	0.015	0.012	0.022	0.015	0.01	0.02	0.900
Total bilirubin (mg%mL)	0.027	0.022	0.037	0.027	0.02	0.034	0.586
Creatinine (mg%mL)	1.063	0.76	1.637	0.83	0.695	1.16	0.019
CPK (mg%mL)	92.0	46.0	165.5	72.5	41	145.0	0.406
Sodium (mg%mL)	140.0	138.0	142.0	139.0	137	141.0	0.014
Potassium (mg%mL)	4.0	3.6	4.275	4.1	3.8	4.5	0.104
D-dimers (mg%L)	0.771	0.528	2.265	0.908	0.502	1.962	0.882
INR	1.12	1.052	1.202	1.04	1.0	1.09	<0.001
IL-6 (pg%mL)	38.3	17.3	123.0	29.2	7.05	83.5	0.183
IgM(g%L) AU/mL	0.68	0.07	8.842	4.19	0.473	13.303	0.032
IgG(g%L) AU/mL	0.3	0.065	3.955	2.51	0.185	5.74	0.023
Length of stay (days)	11.0	5.75	15	9.0	5.0	16.0	0.837

**Table 3:** Fitting parameters of our decision tree model verified on the validation dataset.

Parameter	Value (%)
Accuracy	75.93%
Sensitivity	99.61%
Specificity	23.43%
PPV	82.18%
NPV	40.07%
F1-score	89.17%
MCC	17.94%
Balanced accuracy	61.52%

## Data Availability

Restrictions apply to the availability of these data.
